# An ethnographic study of Latino preschool children's oral health in rural California: Intersections among family, community, provider and regulatory sectors

**DOI:** 10.1186/1472-6831-8-8

**Published:** 2008-03-31

**Authors:** Judith C Barker, Sarah B Horton

**Affiliations:** 1 Department of Anthropology, History & Social Medicine and Center to Address Disparities in Children's Oral Health, University of California San Francisco, 3333 California Street, Suite 485, San Francisco, CA 94143-0850, USA; 2 Department of Preventive & Restorative Dental Sciences and Department of Anthropology, History & Social Medicine and Center to Address Disparities in Children's Oral Health, University of California San Francisco, 3333 California Street, Suite 485, San Francisco, CA 94143-0850, USA

## Abstract

**Background:**

Latino children experience a higher prevalence of caries than do children in any other racial/ethnic group in the US. This paper examines the intersections among four societal sectors or contexts of care which contribute to oral health disparities for low-income, preschool Latino^1 ^children in rural California.

**Methods:**

Findings are reported from an ethnographic investigation, conducted in 2005–2006, of family, community, professional/dental and policy/regulatory sectors or contexts of care that play central roles in creating or sustaining low income, rural children's poor oral health status. The study community of around 9,000 people, predominantly of Mexican-American origin, was located in California's agricultural Central Valley. Observations in homes, community facilities, and dental offices within the region were supplemented by in-depth interviews with 30 key informants (such as dental professionals, health educators, child welfare agents, clinic administrators and regulatory agents) and 47 primary caregivers (mothers) of children at least one of whom was under 6 years of age.

**Results:**

Caregivers did not always recognize visible signs of caries among their children, nor respond quickly unless children also complained of pain. Fluctuating seasonal eligibility for public health insurance intersected with limited community infrastructure and civic amenities, including lack of public transportation, to create difficulties in access to care. The non-fluoridated municipal water supply is not widely consumed because of fears about pesticide pollution. If the dentist brought children into the clinic for multiple visits, this caused the accompanying parent hardship and occasionally resulted in the loss of his or her job. Few general dentists had received specific training in how to handle young patients. Children's dental fear and poor provider-parent communication were exacerbated by a scarcity of dentists willing to serve rural low-income populations. Stringent state fiscal reimbursement policies further complicated the situation.

**Conclusion:**

Several societal sectors or contexts of care significantly intersected to produce or sustain poor oral health care for children. Parental beliefs and practices, leading for example to delay in seeking care, were compounded by lack of key community or economic resources, and the organization and delivery of professional dental services. In the context of state-mandated policies and procedures, these all worked to militate against children receiving timely care that would considerably reduce oral health disparities among this highly disadvantaged population.

## Background

The Latino immigrant population is the fastest-growing and largest minority group in the United States (US), numbering 35.3 million in the 2000 census [1]. Of the approximately 4.2 million farmworkers and their dependents in the US, over 80% self-identify as Latino, with 75% of Mexican origin [2]. Of all population groups, migrant and agricultural workers have the greatest difficulties accessing health care [3,4], especially oral health care [5,6]. Oral health problems are highly prevalent among migrant populations [7,8].

Dental caries is the most common chronic and infectious childhood illness [9]. During the California Smile Survey, conducted in 2005, over 21,000 kindergarten and 3^rd ^grade students were assessed with comprehensive dental screenings. Over half of the children examined were Latino – 72% had some caries, while 26% had rampant caries in seven or more teeth. These results were nearly twice the determined rates for the non-Hispanic white population [10]. Young Latino children, those age five or under, have higher rates of early childhood caries (ECC) than any other ethnic/racial group. Mexican-Americans as a subgroup have the poorest oral health status among Latinos [9-13]. Rates of ECC are highest among migrant children [10,11]. In several regions in the US, including North Carolina and Southwest Virginia [5], Michigan [14,15], Southern Illinois [16], Colorado [17], the Yakima Valley in Washington [18] and the Central Valley in California [19], children of Mexican-American farmworkers have been noted as having especially poor oral health.

Despite this need for dental care, Latinos of all ages have the lowest dental utilization rate of all ethnic/racial groups, with Mexican-Americans having the lowest utilization rate of all Latino groups [12,20]. These findings persist even after controlling for factors such as age, income, education, sex and dental insurance coverage [21]. The 2000–2003 National Health Interview Survey reported that 16.7 percent of Latino children ages 2–17 years, and 17.7 percent of Mexican American children, had never seen a dentist [22]. Wall and Brown [21] also found that Mexican-Americans were two to three times more likely to visit a dentist than were Mexican immigrants born in Mexico but living in the United States.

Research has documented various barriers to access to and utilization of health care, including dental care, for children in low-income Mexican-origin populations in the US, such as lack of health insurance, transportation, limited English language proficiency, and so forth [8,19,23-25]. A major focus within the literature has been on parental beliefs and behaviors, on discerning when and why parents do or do not seek dental care for their children [26-29]. While caregiver attitudes and practices are important and undoubtedly affect children's oral health and access to care, these are not the only things that do so. The characteristics of specific communities in particular locations and the civic services and resources available, staff attitudes and practices within dental clinics, financing mechanisms and the workings of the public health care system, constitute other contexts which all directly or indirectly affect dental care and so also contribute to the creation and maintenance of children's oral health status. Thus, research which focuses on caregivers alone gives just part of the picture, examines just one context of care.

Several conceptual models point to multiple societal levels or contexts of care influencing children's oral health [30-32]. The most well-known conceptual model is Stokols' Social Ecological framework [32] which posits that (dental) disease in young children is an outcome of interaction among intraindividual, interpersonal, institutional, community and social/policy contexts. Stokols' intraindividual level was not directly accessible because we could not interview preschool children about their habits, behaviors, skills, knowledge, attitudes, beliefs, or fears. So we collapsed Stokols' intraindividual and interpersonal contexts together into what we called the Child/parent/family context. To date, very little empirical research has actually explored the intersections among these various contexts of care, especially intersections that collectively contribute to low dental utilization rates and poor oral health status for Latino children. The ethnographic study presented here, however, does exactly that. It examines major contexts of care and demonstrates how these intersect synergistically helping influence oral health status for young Latino children in a rural California community. The four contexts we examine are (**1**) Child/parent/family context; (**2**) Community and civic context; (**3**) Professional and oral health delivery context; and (**4) **Policy, regulatory, and public finance context.

## Methods

### Ethnography

Ethnography – a combination of community mapping, participant-observation and in-depth interviews – is a flexible yet rigorous and systematic style of qualitative research, with well established conventions for data collection and analysis [33,34]. The theoretical stance underpinning this qualitative research is constructivist, consistent with grounded theory procedures [35,36]. Firsthand accounts from knowledgeable individuals situated in various contexts are used to develop, first, an understanding of the meanings people give to their experiences and the sense they make of seeking, delivering or managing oral health care services for low-income Latino children. Moreover, these accounts can help discern how these meanings travel across the boundaries of these contexts. Ethnography's holistic approach further allows for an examination of the interaction among multiple agents in the various contexts, creating a more complex and nuanced picture [37,38].

Ethnography aims at understanding the life-world of people in particular communities from their points of view, and at revealing the complex interactions and connections among the various contexts that frame the opportunities and constraints individuals encounter. An advantage over other research approaches is that as fieldworkers live and work in the community under study for an extended period of time, they are able to identify various contexts and pursue connections among them as they become apparent through participation in and observation of the everyday routines of community members. These observations and experiences are confirmed and given further shape and depth through focused probing interviews with central, influential figures. Ethnographic analysis portrays the health care system and its interconnections with patients' lives and activities, community resources, and policy contexts, systematically capturing issues that may not be apparent in interviews or observations focused solely on one respondent group or one context alone. It captures *the processes *through which disparities in children's oral health unfold. It engages in "thick description," examining first, for example, caregiver beliefs and behaviors and the meanings they give to children's oral health, and then following along as these meanings serve to guide the caregivers' interactions with the health care system. In this process, these meanings become nuanced in different ways, come to take on new forms, or develop additional meaning. By triangulating and comparing data gathered in multiple ways over a nine month period from a variety of sources – dentists, other health professionals, agency and community officials, early childhood educators, and school nurses, along with information obtained from parents and from dental advocacy organizations and state officials responsible for interpreting policies and administering reimbursement – this ethnography offers a rich portrait of the multi-factorial causes of the poor oral health status of impoverished, rural Latino children.

Although they are important factors and the primary focus of many clinical studies, individual biologic/physiologic contributions to children's oral health [39] are not included in this discussion precisely because ethnography seeks to understand how factors at the level of family organization, culture, community and societal institution operate to affect health status. Based on our previous research experience and our reading of the existing literature, four contexts were identified as key to the oral health outcomes of Latino children, and formed the basic foci around which ethnography was conducted. These contexts, and their component parts that guided our inquiry, were:

• *Child/Parent/Family Context *– comprises the nature, range and diversity of parental knowledge and beliefs about oral health and dental care needs for children, especially children from birth through five years of age. This context included: the causes and symptoms of oral disease, beliefs about treatment, help-seeking attitudes regarding dental problems; children's behaviors and kinds of care practices undertaken in the home with respect to children's oral health (such as use of bedtime bottles containing sweet liquid, oral hygiene, diet); the caregiver's own experience of oral heath care as a child and perceptions of difference from the lives of their children; caregiver accounts of and general comments about access to and use of oral health care services in the region. Family characteristics, such as health insurance status, occupation, migrant status, language preference, education and income level, and the impact of these on access to and use of services for children's oral health problems, are also key. These characteristics result in part from individual beliefs or actions and in part from a family's affiliation with a specific population group and its social position in the local community and thus its ability to access resources.

• *Community and Civic Context *– encompasses various life and socio-economic circumstances of community residents and the interface of these circumstances with oral health. Characteristics of specific communities – such as their particular regional associations; rural, suburban or urban locations; number and type s of resident; the nature and diversity of occupational opportunities; and the availability and types of civic amenities or resources – can all influence oral health status. Some of these interact directly with access to or support oral care, such as health centers, transport to dental services, or fluoridation of public water supply. Other community services or facilities have a more indirect influence, such as libraries, job training sites, the presence of a range of health-related businesses, such as pharmacies.

• *Professional and Oral Health Delivery Context *– the sector most obviously connected directly to the oral health status of children is that concerning dental practice, especially the number and distribution of dental clinics in the locale, and their capacity to provide both general and specialty care for young children. Characteristics of providers and clinic staff and how provider practices affect Latino children and families are also central aspects of this sector – whether, for example, staff members speak Spanish; the facilities offer services on weekends or evenings; the clinic accommodates low-income patients through a sliding fee scale; parents are permitted to remain in the room when their children are being examined or treated.

• *Policy, Regulatory, and Public Finance Context *– state dental health policy and fiscal regulations and reimbursement practices also have an impact. Rural and urban dental clinics, for example, face different reimbursement guidelines, concerning the type of evidence a dentist has to submit to substantiate payment requests. Oral health providers working in private and public clinics experience different regulatory and fiscal constraints that intersect with their practice preferences regarding treatment and prevention. Again, these affect children's oral health status.

### Study location

The study, conducted in 2005–2006, focused on both recent migrants and longer-term residents of a small agricultural town in Fresno County in the San Joaquin or Central Valley of California. Around 95% of the permanently resident population of approximately 9,000 is of Latino, largely Mexican, origin. The town's population is young, with a median age of 25.4 years and one-third under 18 years of age [40]. Agriculture, based around the production and distribution of cantaloupes, tomatoes, peaches, almonds, asparagus, and broccoli, is the main economic enterprise in the region providing employment to the majority of adults in the town. This economic base results in marked seasonal fluctuation in the town's population size, household income and health insurance status, as well as a high overall poverty rate; some 40% of the 1,825 households in the city live at or below federal poverty level, currently defined as an annual income of $US24,000 for a family with three children [41]. Average family size was 4.3 in 2000 [40]. While very recent housing construction, including federally-subsidized developments, have provided accommodations of a good standard, many farmworker families still live in sub-standard, crowded dwellings. Monthly rental payments of $1,000 per house were common, making it necessary for several families to share a single home. The city hosts three small supermarkets, two large elementary schools, a high school, both regular and migrant-focused early childhood education facilities (Head Start), an agency of a federal nutrition program (Women, Infants and Children [WIC]), and a Federally Qualified Health Center providing primary care, very basic medical emergency services, and dental care. In addition, there are two other private general dentists in the town itself, with another 20 dental offices located approximately within a 45-mile radius. We included in this study all eight of the dental offices within that general radius that accepted pediatric patients with Denti-Cal, the state's public dental Medicaid program, as well as several other private dental offices.

Before commencing any research activity, study protocols and procedures were first approved by the University of California San Francisco's Institutional Review Board. In addition to informal conversations and observations at doctor's or dentists' offices, community, civic and government agencies, shops, schools, cafes, homes, churches and community events, contact was made with two kinds of formal study subjects: key informants and caregivers. Both were formally interviewed using a semi-structured in-depth interview guide and probe questions as needed. All formally interviewed research participants provided written informed consent. As compensation for their time and expertise, a small honorarium was provided ($20 store coupon per interview occasion for caregivers, or $50 donation to a charity of their choice for key informants including health professionals).

Topics in the semi-structured interview guides used with caregivers and key informants were based on previous studies of Mexican immigrant and low-income populations' conceptions of oral disease and experiences with the (oral) health care system [8,14,15,18,19,23-26,29,42-46]. Questions also were to answer the main ethnographic research agenda – namely: (**a**) to document the nature, range and diversity of knowledge and beliefs about oral health and dental care needs for children from birth to five years of age in this rural Latino population; (**b) **to understand how these beliefs related to the kinds of dental care practices undertaken in the home with respect to children, such as use of bedtime bottles, or tooth brushing; (**c**) to uncover the relationship between beliefs and practices and (i) expressed willingness and (ii) demonstrated ability of caregivers to seek and use oral health services for young children; (**d**) to investigate the impact of life circumstances of parents (eg, insurance, migrant status, language preference, etc) and barriers and facilitators to their seeking and using care for children's oral health; and (**e**) to document how well – and why/why not – preventative messages and advice given by professionals and dental service personnel, especially about ECC, are (i) accepted and (ii) incorporated into oral health practices for young children. The interview guides were developed in consultation with a team of specialists in Latino children's oral health, including a pediatric dentist and dental public health researcher as well as a specialist in Latino family issues from the University of California at Davis. Interviews were conducted until data saturation was assured with no new information forthcoming.

Data analysis followed standard techniques for thematic identification and coding [33-36]. Codes were developed from two sources; first (pre-codes) from the initial research questions underpinning the study, and second (post-codes) from the text itself. At least two people independently read through fieldnotes and transcripts of interviews, noting ideas and commonalities as they emerged. Once developed, codes were systematically applied to the text, with coded text segments independently checked to ensure consensus and consistency. Initial codes were subsequently refined and coding continued in an iterative fashion until further meaningful sub-divisions could not be discerned in the text. While many themes were discerned at varying levels of specificity, those reported below are the major ones associated with each context.

### Interviews: Key informants

Key informants, while not the main focus of a study, have relevant knowledge or insight into the issues of interest, and provide a perspective important for properly contextualizing observational data and the comments of study participants. Overall, 30 key informants were included in this study. Three major kinds of key informant were included: ***(A) ***– child health educators and nutritionists who interacted with caregivers and provided information about oral health, including: WIC educators and nutritionists, Head Start directors and teachers, and school nurses; ***(B) ***– officials from the county's dental advocacy organization and state officials responsible for administering Denti-Cal policy and reimbursements, who discussed the impact of state welfare and fiscal policies on the access to care and the oral health status of these low-income rural children; and, ***(C) ***– health professionals in the region, including dentists and physicians, who discussed their experiences with Latino children and their views of oral health care. More informal interviews were also done with a diverse set of local community leaders gathering background information on the structure of local government and service availability. Key informants were asked to describe generally (a) their professional role as it touched in any way on health, and to discuss (b) the oral health status of Latino children in the community, including their views on and ability to affect the cause, circumstance or outcome of children's oral heath.

### Interviews: Caregivers

The majority of formal in-depth interviews conducted, however, was with caregivers of young children. Eligible participants were: **(1) **primary caregivers aged 18 or older who provided regular daily care for at least one child under the age of 5 (e.g., either parent of the child, a grandparent, aunt, day care provider); and **(2) **immigrants from Latin America (both Mexico and Central America) or first-generation Mexican-Americans born in the United States. Selection was not based on whether or not the caregiver's child had experienced caries or had ever been to a dentist. Caregivers were recruited through two sources: some 2/3^rds ^(around 30 people) came from a listing of a randomized sample of farmworker household addresses generated by a partner epidemiologic study on farmworker occupational health, conducted by the University of California at Davis; around 1/3^rd ^(some 17 people) came from two local Head Start programs with high Latino enrollment. Interested participants were screened for eligibility and recruited into the study by two bilingual interview staff, who obtained written informed consent. Each of the 47 participants was interviewed at least once, usually in their homes; several were interviewed up to three times (a total of 80 interview occasions). Each interview lasted between 11/2 -2 hours. Participants received a $20 gift certificate to a local grocery store for a first interview, and a $10 certificate for each subsequent interview.

Through a series of open-ended questions, caregivers were asked to describe their backgrounds, present socio-demographic circumstances, their own experiences with oral health care over their lifetime, their children's experiences with oral health, and their ideas about oral health, daily practices around oral hygiene and diet as well as experiences with preventing and seeking treatment for their children's oral health. The interview focused on the oral health experience of the caregiver's child nearest in age to 4 or 5 years, including as appropriate contrasts and comparisons to the oral health experiences of that child's siblings. As necessary, each caregiver was asked follow-up and probe questions to ensure that as complete an account as possible was generated during the conversation.

### Observations

Documentation was made of the pace and nature of daily life, and seasonal variations in the rhythm of communal activities over the nine month intensive study period. In addition to oral-health related observations made during interviews or visits to the homes of caregivers (of 15 caregivers and their children during snack-time, often involving feeding children a baby bottle; and of 5 caregivers and children during a meal-time), the fieldworker (SBH) also made extensive observations of interactions among staff, parents and child-patients at the front desk/waiting area in eight different dental offices in the wider region. Written fieldnotes about these observations included location, time of day, persons present, languages used during interaction, topics discussed, duration and nature of information imparted or written materials exchanged, activities undertaken or tasks performed. On five occasions, SBH also provided transportation, accompanying families throughout their entire visit to dental offices when seeking care for their children. These extensive observations included trips to pediatric dental specialists, all of whom were located 45 miles away. A week was also spent closely observing work in a county-provided mobile dental van that was stationed in the city for three months providing urgent care for third-graders (8 year olds) in one of the city's elementary schools.

## Results

### Sample: Key informants

Interviews with key informants provided insight into the ways in which the community, oral health delivery and state regulatory contexts of care intersected with each other and overlapped with the family caregiver context. Central among these 30 key informants were the 12 dentists working within a 45-mile radius of the city in which the study was conducted. These oral health professionals proved crucial to the investigation. Dentists commented about their provision of care and interactions with family and about the ways in which the state regulatory context shaped and constrained their ability to offer children certain kinds of care.

The main socio-demographic characteristics of these health professionals and their practices are provided in Table 1. The four dentists in private practices had distinctly different characteristics than the eight dentists working in public clinics. (Because the distribution of responses is skewed, we report here both medians and means). Those in private practice were: white; predominantly dental specialists; located at the periphery of the 45 mile circle centered on the study site; practicing a long time (average 37 ± 6 years, median 37, range 30–45); and, working in their present location for more than a decade (average of 20 ± 7 years, median 15, range 15–30). In contrast, only one of the dentists in the public clinics was white, and none claimed a dental specialty. Compared to those in private practice, this group were located closer to the study site, had been in practice for a considerably shorter period (on average 8 ± 6 years; median 2, range 0.5–18), and had been located in the region for a far shorter time (on average 3.5 ± 5 years, median 2, range 0.5 – 15). Despite these notable demographic differences between the dentists working in private and public settings, there were not markedly consistent differences in their viewpoints about their young Latino patients' oral health status or habits.

**Table 1:** Characteristics of dentists interviewed (n = 12)

**Gender**	
Male	11
Female	1
**Race/Ethnicity**	
White	5
Latino	2
Asian	5
**Practice Location**	
Rural setting	9
Urban setting	3
**Dental Practice Type**	
General Dentistry	9
Specialist*	3
**Practice Setting**	
Private Practice	4
Public Clinic	8
**Accepts Denti-Cal (Dental Medicaid)**	
Yes	11
No	1
Years in Practice**	
Average	16.9 ± 14.6
Median	17
Range	0.5 – 45
< 1	1
1–5	2
6–20	4
20+	3
Years practicing in Present Location**	
Average	8.7 ± 9.2
Median	3
Range	0.5 – 30
< 1	1
1–5	5
6–20	3
20+	1

All dentists accepted children under age six as patients, and were located within a 45-mile radius of study site.

* In pediatric dentistry or oral surgery

** Total number of respondents for this question was 10

### Sample: Caregivers

Socio-demographic characteristics of the 47 caregivers, who provided the bulk of the information pertinent to the family/parental context of care, are given in Table 2. In terms of age, household and family composition, educational levels, income, and occupation, this sample broadly matches the 2006 census data reported for the city [40]. Though we set out with a broad definition of caregiver, the sample turned out to be very homogeneous.

**Table 2:** Socio-demographic characteristics of rural Latino caregivers

Total Respondents*	**Mexican**	Central American**	**US-Born Mexican American**	**All Caregivers**
	(n = 26)	(n = 14)	(n = 7)	(n = 47)
**Gender**	n = 26	n = 14	n = 7	n = 47
Female	26	14	7	47
**Age**	n = 25	n = 13	n = 7	n = 45
Mean ± SD	30.4 ± 6.2	32.4 ± 6.4	27.0 ± 5.5	30.5 ± 6.5
Median	29	32	27	29
Range	19–47	24–45	20–35	19–47
< 20 years	1	0	2	3
21- 30 years	15	6	3	24
31+ years	9	7	2	18
**Education Completed**	n = 25	n = 14	n = 7	n = 46
Mean ± SD	7.1 ± 3.7	3.6 ± 2.8	12.2 ± 0.7	6.8 ± 4.2
Median	9	2.5	12	6
Range	0–14	0–10	12–14	0–14
0–3 years	6	8	0	14
4–6 years	5	5	0	10
7–9 years	10	0	0	10
10 – 12 years	2	1		9
13+ years	2	0		3
Annual Household Income***	n = 24	n = 14	n = 6	n = 44
Mean ± SD	$17,0000 ± 5,700	$17,000 ± 7,500	$23,000 ± 13,500	$17,5000 ± 8,000
Median	17,500	12,000	22,000	$16,000
Range	$8,000 – 28,000	$8,000–36,000	$6,000–50,000	$6,000 – 50,000
< $10,000	4	3	1	8
$11,000 – 15,000	6	5	1	12
$16,000 – 20,000	9	2	1	12
$ 21,000+	5	4	3	12
**Marital/Partner Status**	n = 25	n = 14	n = 7	n = 46
Mother has partner	24	14	6	44
Mother is single	1	0	1	2
**Years in US**†		n = 25	n = 14		n = 39
Mean ± SD	8.5 ± 5.6	10.2 ± 3.5		9.0 ± 4.0
Median	7	10		9
Range	3–22	5–18		3–22
< 10 years of residence	18	8		26
10+ years of residence	7	6		13
**Legal Status**†	n = 25	n = 14	n = 7	n = 46
Undocumented	17	5		22
Temporary Permanent Status	0	5		5
Asylum	0	1		1
Legal Permanent Resident	7	3		10
Citizen	1	0	7	8
**Occupation**	n = 24	n = 12	n = 7	n = 45
Farm work	13	12	0	25
Full-time Caregiver	10	0	7	17
Other	1	0	0	1
**Rural or Urban Origin**§	N = 26	n = 12	n = 7	n = 47
Rural origin	21	10	7	40
Urban origin	5	2	0	7
**Children per Household**	n = 26	n = 14	n = 7	n = 47
Mean ± SD	2.7 ± 1.2	2.5 ± 1.2	2.6 ± 1.4	2.7 ± 1.2
Median	3	2	2	2
Range	1–5	1–5	1–5	1–5
1 child	4	3	2	9
2 children	7	5	2	14
3 children	10	4	0	14
4 children	2	0	2	4
5 children	3	2	1	6
**Age of Youngest Child**	n = 25	n = 12	n = 7	n = 44
Mean ± SD	2.3 ± 1.4	2.9 ± 2.0	2.7 ± 1.8	2.6 ± 1.6
Median	2	2.5	2	2
Range	2 weeks – 4 years	6 months – 6 years	3 months – 6 years	2 weeks – 6 years
< 1 year	6	2	2	10
1–2 years	8	4	2	14
3–4 years	11	4	2	17
5+ years	0	2	1	3
**Age of Oldest Child**△	n = 23	n = 10	n = 5	n = 38
Mean ± SD	10.2 ± 5.7	9.6 ± 5.6	9.4 ± 4.1	9.9. ± 5.4
Median	10	8.5	9	9
Range	2 – 24	3 – 19	3.5 – 14	2–24
< 5 years	6	3	1	10
6–10 years	9	3	4	16
11+ years	8	4	0	12

Because the distribution of responses is skewed, where applicable, both means and medians are provided.

*The number of respondents varied slightly by question; so number of replies is noted for each item.

** Of the 14 Central American study respondents, 12 (86%) came from El Salvador; one had arrived from Honduras, one from Guatemala.

*** In $US 2006.

† Length of residence in US not applicable to US-born citizens; legal status other than citizen is not applicable to US-born Mexican-Americans.

§A rural town was defined as having a population of 15,000 or less. An urban area was defined as having a population larger than 15,000. Despite their larger size, these urban areas in Mexico were not major metropolitan areas but rather central communities primarily serving the surrounding rural region.

△ When more than one child lived in a household.

These primary caregivers, all Spanish-speaking mothers, were on average 30 years of age with three children, the majority of whom were under five years of age. In general, the women had completed school only through 6^th ^grade, although education varied markedly by national origin and location (i.e., rural or urban origin) in their home countries. US-born citizens had all completed high school, whereas several Central American migrants had received no formal schooling at all. The majority of immigrant caregivers were undocumented (i.e., illegally present in the US) and had been in the United States for less than 10 years. Neither length of residence in the US nor educational attainment were reflected in incomes as just as many of the poorest and the wealthiest study participants had been in the US a short time or a long time, or to have completed far fewer or far more than the average years of schooling. Just over half (53%) of these caregivers were employed full- or part-time in farmwork. Full-time caregiver/homemakers had husbands employed in agricultural occupations. Overall, only 20% (n = 9) of women in the sample lived in families with an income that matched or exceeded the federal poverty level for 2006, with only two individuals residing in households making $30,000 or more annually. This reflects a level of poverty that makes paying out-of-pocket for dental services extremely difficult if not prohibitive. As a group, caregivers had had significant experience with children's oral disease: for example, of the 38 immigrant Mexican or Salvadoran caregivers, 23 (60%) reported that their focal child under age six had had cavities; eight reported their child had no dental visit yet and seven reported their child had had a visit but no cavities yet [47,48].

Socio-demographic variation in the sample characteristics (such as caregivers' income, education, length of time in US, origin in a rural or urban area, and country of origin), led to very few discernible differences in espoused dental health beliefs or practices. The oral health experiences of the preschool children, as reported by these women, are remarkably similar.

### Intersecting contexts of care

Reported here are the most prominent of the numerous themes that emerged from the text and observational data relevant to each societal sector or context of care. These examples illuminate the direct outcomes, in terms of young Latino children's oral health status, of intersections among the four identified contexts of care.

Many of the factors discussed here had previously been identified as important in other studies [5,9,12,19,24-30,43-46]. We do not merely replicate those results, however, but demonstrate how these various robust findings inter-relate dynamically. Items in one context shift from, travel across boundaries, and generate a different but related effect in other contexts. Every finding is made complex and multi-faceted through its synergistic intersection with and re-emergence in other contexts of care. As an example, we examine and trace the concept of 'delay,' from context to context. Delay in accessing care occurs for several reasons – because of various ideas or actions (or inactions) of parents or of professional dentists or because of the opportunities (or lack thereof) and constraints/barriers presented by social, community, economic, legal or policy circumstances. Notice how 'delay' maneuvers across and weaves its way through these four contexts, adding new layers of meaning and becoming manifest in various guises, easily spinning out of the control of any particular person or context of care. Observe how all these instances of delay serve to exacerbate a lack of timely access to completed treatment. In another example, in Context 3, that of professional (dental) health care practices, important influences are that most dentists do not speak Spanish and that they serve rural clinics for short periods of time. As a result, dentists often do not develop a deep commitment to the constituents they serve. This finding appears in a different but related form in Context 1, that of families/caregivers, when parents talk about not knowing who the dentists are, of not trusting providers with whom they cannot speak directly and whom they suspect of disrespecting them. Because they straddle between the domestic or family context of care and the state regulatory context, dentists reveal not just the intersections between those two contexts but the dynamic, malleable, multi-directional nature of those connections.

### Context of care (1) – Child/parent/family context

Latino parents wanted their children to be healthy. They were very eager and willing to improve their children's oral health status but often lacked the knowledge, resources or official assistance to do so effectively. While parents desired their children to have "healthy", strong, "white teeth", they were often inconsistent in teaching and supervising their children with respect to good oral hygiene practices. Very few understood caries to involve an infectious process. On occasion, children shared food or eating utensils with each other or their parents, or toys and baby bottles were exchanged between infants. Some very low income parents reported the cost of giving each child in the family their own toothbrush was an expense they could not afford.

Rural, immigrant parents tended not to recognize dental caries in their children as a disease, but rather classified visible discoloration on teeth due to cavities as "stains" ("*manchas*") and tended not to seek help for 'stains' on children's teeth – to delay seeking care – unless these were accompanied fairly consistently by swelling and or complaints of pain [47]. Because tooth decay is viewed as a "stain", when treatment is sought, parents often requested and expected a "cleaning" *("limpianza")*. The need for, and associated costs, of restorative work therefore came as an unpleasant surprise. Mothers accepted being educated about "baby bottle decay" by knowledgeable sources such as WIC, but frequently misunderstood the messages being delivered. For example, mothers spoke of the bottle's teat or nipple as the problem, not the sweet fluid content of the bottle [48].

Many recent migrant mothers often claimed that they themselves had never had dental treatment, even as an adult. Many immigrant parents who had never experienced cavities when young but now have children with high rates of dental caries were puzzled by this disease, unsure of how to prevent it or treat it. Migrant parents also reported major changes in diet since moving to the US, and commented on how different their children's diet is from their own when growing up. Major differences concerned their children's greater consumption of sugar (candies), sodas and lesser access to fresh vegetables and fruits. Parents did not specifically associate these broad dietary changes with their children's dental problems although they did connect consumption of sweet substances with the subsequent advent of caries [47,48]. Collectively, all these understandings and actions on the part of caregivers set up rural Latino children, especially the children of recent immigrants, for a high rate of unrecognized and untreated oral disease.

### Context of care (2) – Community and civic context

In the majority of farmworker families, parental income, and thus eligibility for public health insurance, fluctuated seasonally in concert with the agricultural crop cycle from planting to harvest. Parents were not always aware of exactly when or whether their children were eligible for services paid for through public 'safety net' monies (i.e., Denti-Cal). During harvest season particularly, parents worked long hours with inflexible schedules. Often, they were unable to get time off work to accompany their child to a medical clinic or dental office, especially if the child needed recurrent visits. Some parents reported losing their jobs because of a need to repeatedly take a child for health care, usually dental care, within a very short period of time. As a result, because of uncertain eligibility for Denti-Cal and difficulty in getting time off work some parents delayed seeking care for their children's oral health problems. Parents tended to wait to take their children to a dentist until they were semi- or unemployed in the winter months, when their income was lowest and they knew their children would definitely be eligible for Denti-Cal services and they had time to accompany children to the clinic.

The public water supply in the research site was not fluoridated. Moreover, a recent history of problems with the water – such as turbidity, discoloration and possible pesticide pollution – made parents wary of using municipal tap water for drinking or cooking. Avoidance of public water supplies by Latino communities has been noted elsewhere, too [49,50]. Instead, at the study site, non-fluordiated water was purchased in bottles or from commercial filtration sources known locally as "water mills." This lack of access to a safe, fluoridated municipal water supply deprived children of ready access to a proven effective means for preventing caries. And this preference for water purchased commercially strained already slim economic resources within a family.

Lack of public transportation delayed and limited access to distant specialist health care services. Many of these immigrant women did not drive, and most families did not own a vehicle. Private transportation was often difficult to arrange and expensive for families. Parents who could not afford to pay privately for preventive or restorative treatments, or could not find time to repeatedly take a child to the dental clinic, occasionally waited until making a visit to Mexico to get their children care at much lower costs. Or they resorted to extraction of affected teeth as an affordable procedure.

For all these community/civic-based reasons, then, the children of farmworkers did not receive recommended regular checkups and timely dental visits. These constitute reasons beyond the family context why oral disease went largely unrecognized or untreated in farmworker children.

### Context of care (3): Professional and oral health delivery context

Educational and social welfare agencies, such as the early childhood education organization Head Start and federal Women Infant and Children (WIC) nutrition programs, play an important role in educating parents about oral health, and in introducing children to dental hygiene self-care practices such as tooth brushing [51,52]. While local physicians and others often recommended that young children receive dental care, staff at Head Start and WIC were the people with whom parents interacted most frequently. These professionals repeatedly urged caregivers to take children to dental professional for care. Many caregivers reported taking – or trying to take – their children for a first dental visit because of the encouragement provided by these community-based resources. Despite this, many children aged five or under experienced a need for extensive dental treatment, a need that for many children remained unmet.

Parental delay in seeking care for children, an action rooted in Contexts 1 and 2, was compounded by a fundamental lack of access to basic oral health care. Few dental clinics were willing to treat young children, especially those under four years of age, and fewer still accepted Denti-Cal reimbursement, making it difficult for low-income Latino parents to find services for young children or to get them timely care. Of 23 dental clinics in the region circumscribed by a 45-mile radius from the study site, only eight (34%) accepted as patients children under age four on Denti-Cal. This resulted in long waiting lists at these clinics and months of delay until children could actually be seen. Practitioners' preference to not serve young children was bolstered by the fact that many general dentists spoke of feeling uncomfortable and unprepared to deal with young children's behavioral issues, citing only a minimal or total lack of training about handling pediatric patients while in dental school. General dentists were often not familiar with currently acceptable techniques for working with children who cry, scream, squirm or resist examination or treatment.

For some children, a lengthy delay in accessing care seriously exacerbated their need for oral health treatment and ended in referral to a specialist clinic because of the urgency or extensiveness of treatment needed or the necessity for care to be performed under general anesthesia, a far riskier procedure for children than ordinary dental procedures. Within this region, however, there were only three specialist clinics that treated Denti-Cal pediatric patients needing advanced care. Because of their scarcity, pediatric specialists had waiting lists ranging from 2 to 5 months, so children experienced further delay in receiving care. Typically, the pediatric specialists saw Denti-Cal patients just one day a week in a type of 'assembly-line' fashion that provided treatment quickly and efficiently but very impersonally to as large a number of children as possible. Specialty clinics were located in urban areas 45 miles distant from the study site, making physical access difficult given the absence of public transportation. Whereas private paying or privately-insured patients were seen far more quickly at both general and specialist clinics, children on Denti-Cal had fewer choices.

Few parents spoke more than rudimentary English; fewer still were literate in either English or Spanish. Monolingual Spanish-speaking or illiterate parents did not understand official forms written in English and so relied extensively on bilingual staff at health clinics or social service agencies to fill in paperwork and facilitate access to care for their children. When agency workers could not communicate in Spanish – or did not bother to do so when they were able- this barrier was magnified. Although most dental offices in the region had Spanish speakers on staff, these personnel were not always trained in or skilled at health care translation or at health education/promotion work. Moreover, staff members were often very busy and did not have time to explain fully the nature of the paperwork or the treatment plan. Hence, parents frequently did not know exactly what dental services they were agreeing to for their children, nor did they understand the inherent risks of certain procedures (e.g., general anesthesia). Despite these uncertainties, parents agreed to treatment because they wanted their children to receive care. Few of the dentists themselves spoke Spanish. Hence, direct communication or health education between parents and dental professionals was limited, and mediated by staff or family members with unknown levels of skill or knowledge in translating biomedical or technical information.

Language difficulties had several adverse effects. They lowered the level of understanding and trust that parents had for dental health care professionals; some parents reported that they made them feel disrespected or discriminated against. Such language barriers also frustrated providers who were unsure how well parents understood the information being imparted, and unable to assess accurately the level of parental concern over their child's well-being. On one hand, occasionally, especially when delays resulted in a need for extensive or extraordinary rather than routine treatment, this led some providers to indulge in unfortunate and inaccurate stereotyping of Latino families as unconcerned, unwilling or uninterested in accessing care for their child. On the other hand, occasionally, some health care providers had an accurate picture and sympathetic understanding of the many economic and social difficulties Latino farmworker families routinely faced and were as supportive as possible of caregivers' efforts to provide care for their children.

Lack of trust and understanding of health professionals became a particular issue if, as some did, dentists excluded parents from the treatment room while their child was receiving care. Parents were often very angry when children later complained about being strapped down, being yelled at or verbally threatened, having had a hand placed over their mouth to stop crying, or experiencing similar constraints. In response, too, children developed a strong fear of dentistry, which further delayed care seeking or even resulted in the child not returning to the clinic because parents were reluctant to force their child to accept care or complete restorative treatment under such circumstances.

Rural areas are usually medically and dentally underserved [53,54]. As Table 1 demonstrates so clearly, most dentists in the region work in public clinics and have served the area for two years only. Long enough to satisfy their requirements for training and loan repayment programs, but not long enough to develop long-lasting ties with patients or the local communities they serve. One local public clinic was known for its "revolving-door" dentists – in the four years since it began operation, two dentists had already come and gone, and a third had just arrived. People complained about a lack of continuity of care especially for children with extensive on-going need for treatment. The lack of stable dentists in public clinics exacerbates difficulties in the building of trust with local communities.

### Context of care (4): Policy, regulatory, and public finance context

Denti-Cal, the state-funded Medicaid public health insurance program for low-income children [55], has two policies that particularly affect dental practice in rural areas. These practices can deleteriously affect children's oral health status. The first of these policies is that public clinics – Federally Qualified Health Centers (FQHCs), Community Health Centers (CHCs) and Rural Health Centers (RHCs) -which predominate in rural areas, can charge per visit rather than per procedure. This encouraged some dentists to have children return very frequently, often for minimal treatment and for short visits each time. This policy was a strain on caregivers employed in the agricultural sector, and on their ability to organize transportation to and from the clinic. The second policy is that Denti-Cal will only reimburse providers for treatment of cavitated lesions breaching the dento-enamel junction. Until recently, treatment of pre-cavitated white spot lesions with remineralization therapies and preventive treatment, apart from prophylactic fluoride foam treatments every six months, had not been reimbursable. Late in 2006, however, Denti-Cal approved reimbursement for the application of fluoride varnish for preschool age children. Since most dental clinics in this area do not serve children younger than 6 years of age, however, this seemingly positive change might not have much impact.

Many providers, especially those in private rather than public practices, were unable to withstand the economic costs of providing care that Denti-Cal did not reimburse adequately. At the same time, many of these dental providers believed that treatment of pre-cavitated lesions was necessary, especially in the case of Latino children who are at such high risk for dental caries. Due to the lack of reimbursement for such treatment, however, children often did not receive optimal treatment until decay had reached an advanced stage. As a consequence, treatments sometimes necessitated the use of pediatric dental specialists and resort to more expensive and riskier procedures.

Many families were "mixed status" families composed of both documented children (i.e., those born in the US and therefore legal citizens) and undocumented children (i.e., those who were foreign-born and lacked a 'green card', and who were therefore classed as illegal aliens). Federal Medicaid policy permits undocumented non-citizen children to receive a very limited set of services on an emergency basis only. These comprise extractions only and not any preventive or restorative service.

This differentiation by legal status led to some child-citizens not receiving the full benefits to which they are entitled under Denti-Cal. This came about for two major reasons. First, parents greatly fear *la migra*, the immigration authorities. As recently as February 2007, the study site was subject to dawn raids by immigration authorities rounding up illegal migrants in order to deport them. Federal migration policy has a chilling effect at very local levels even on legal citizens. Parents fear the discovery and possible deportation of alien members of the family or of nearby neighbors, co-workers, friends, and the traumatic impact of such disruption on children especially. Second, until the late 1990s, receipt of Medicaid services by citizen-children of undocumented parents was used against migrant parents when seeking naturalization. While the federal government has recently clarified that use of Medicaid benefits by eligible citizen-children should not be grounds for their parents being considered a "public charge," many immigrants do not trust that this is so, and still often avoid health care services for this reason [56,57].

In a setting such as a rural, low-income Latino community, where caries, a highly infectious disease of childhood exists within a high prevalence setting, a discriminatory Denti-Cal policy has only injurious effects. Bacteria do not distinguish the legal status or citizenship of the child's mouth. Not treating undocumented children in a family merely creates a reservoir for continued re-infection and transmission of bacteria to other children resident in the same family, household, neighborhood, school or community.

## Discussion

While few of the specific findings presented above are entirely new or previously unreported, this systematic, broad-ranging ethnographic study of a community has revealed considerable overlap and mutual influence among four contexts of care. Findings and connections among them have to date been largely considered as separate issues. Yet individually and jointly, these intersecting contexts have a synergistic impact on the oral health status of low-income rural Latino children. They contribute both to creating and sustaining oral heath disparities experienced by this already large and still increasing demographic group. These heuristically separable but inextricably interconnected contexts of care comprise: **1**) children/parents/families; **2**) community/civic social sector; **3**) professional and oral health delivery system; and, **4**) public 'safety net' policies and reimbursement regulations.

Specific meanings and actions identified in one context are malleable, dynamic, overlapping and constantly emergent, resonating with each other, crossing contextual boundaries, shifting forms and generating different but related effects elsewhere. In other words, every finding is made complex and multi-faceted through its intricate intersection with and re-emergence in other contexts of care. While each meaning or finding identified in these various contexts can be seen as acting primarily within that context, many items overlap or appear in more than one context, sometimes in slightly altered form. This analysis, however, clearly shows that it is not merely a cultural clash between new immigrant or poorly educated, foreign-language speaking parents and their lack of access to the oral health care system that generates or sustains Latino children's poor oral health status. Rather, the very components of the system militate against children's oral health through the dynamic, sometimes conflicting, often constrictive, usually invisible connections across and within multiple contexts. Some of these connections are out of the control of – and even the awareness of – actors in the other contexts.

Endemic to the practice of dentistry in a low-income rural setting is a particular "culture." Following Good and colleagues [58], we argue that, just as with the "culture of medicine," factors intrinsic to the "culture of dentistry" may create disparities in treatment for ethnic minorities. By "culture" here we mean the organizational settings, opportunities and constraints that constitute, and in turn are further shaped and constituted by, the central practices of a specific group (in this instance, dentists in rural California), as well as the habits, values and viewpoints that are widely shared by members in this group. Here, then, we are referring not to the Latino culture of the study's primary population of interest but rather to the culture of dentistry in this rural region. An analysis of "the culture of dentistry" therefore includes the way dentists in this region interpret, conform to and strategically manipulate Denti-Cal regulations; the values and ideals they learned during their professional training and uphold in their present practices; their beliefs about, connections and commitments to the local community they serve; and, their professional actions, around accepting young children as patients, accepting Denti-Cal payments, controlling the behaviors of young child-patients, interacting with the a child's parents. All these are influential factors in "dental culture."

Constructed within and through the intersection of these various contexts of care, this culture of rural dentistry discourages, even thwarts, caregivers' attempts to access and utilize pediatric oral care services. Dental treatment for low-income children is seriously compromised due to low funding for Denti-Cal as well as a lack of a public health mission among some providers. Children's first experiences with dental providers are crucial in establishing their conceptions about dental care for the rest of their lives. The numbers of providers accepting Denti-Cal, and the rewards for doing so, must be increased, too. Moreover, dental schools should ensure their curricula give greater attention to community dentistry and instill a sense of pride in undertaking dental public health activities, so that dentists do not believe that their responsibilities are limited to the skill of their "clinical hand" but see that their responsibilities are larger than a set of teeth and surrounding oral tissues but, indeed, whole persons in social contexts [39,59]. Greater training for general dentists about pediatric dentistry, especially on how to deal with young children or those with "behavioral issues," could also greatly increase the numbers of general dentists willing to accept small children as patients.

Dental treatment for low-income minority children is also seriously compromised due to low number of individuals from those populations who are being attracted to the dental profession [60], not the least because, compared to dentists from the dominant groups, minority dentists are more likely to work in underserved areas [61]. The startling decrease – a drop of 80% in the period 1983–2000 – in the numbers of Latino dentists practicing in California must be reversed [62]. Although maybe not as dramatically, this decrease is likely to be mirrored elsewhere in the US too, and must be addressed through more aggressive efforts to recruit and retain minority dental students [63].

Because immigrant caregivers lack experience with and knowledge of early childhood caries (ECC), education programs are essential in helping reduce the burden of oral disease. These education programs should take into account caregivers' own inexperience with ECC, explaining the difference between "stains" and cavities, that cavities may be more common in the US due to a more cariogenic diet, that cavities may not always be visible, that early dental checkups are necessary for prevention and to discover early problems, and that nighttime bottle-drinking without subsequent brushing is a problem. WIC agencies and early childhood education services, such as Head Start, are services that can – and do – usefully engage in parental education on dental health issues, but more needs to be done. In addition to referring children for professional oral care when necessary, physician's assistants and other professional staff at these agencies could be trained to do a visual inspection of a child's mouth and teeth and to apply fluoride varnish, a proven caries preventative for children aged 1–5 years [64].

As this ethnographic study was undertaken in just one small, albeit fairly typical Latino city in California's agricultural Central Valley, findings need to be generalized cautiously. Low-income Latino children in urban locations may have somewhat different experiences, as might children from more educated sectors of the rural Latino population, or children from Latino communities with higher socio-economic position. Other low-income children in agricultural communities in California and elsewhere, however, will most likely share many of the characteristics described here and will experience similar barriers to access and use of dental services. It is likely, too, that nationwide, other low-income rural or urban populations dependent on Medicaid funding for access to pediatric dental care have similar experiences. A recent report on the tragic dental-related death of a child in Maryland tends to support these findings [65]. While the specific forms of parental belief and action, community resources and civic amenities, organization of professional practices and state policies, and their points of intersection, will differ from place to place, these four contexts of care will nevertheless operate everywhere in a dynamic, mutually influential fashion. This ethnographic study has thus resulted in a significant expansion of the current literature.

Further research, both qualitative (including ethnographic) and quantitative in approach, should be conducted to discover exactly where and how these contexts synergistically intersect. The current literature too easily encourages an inappropriate "blaming the victim" understanding of low-income and Latino children's poor oral health status by pointing solidly but erroneously to parental socio-demographic characteristics, practices or beliefs as prime causes. While low-income Latino children are victims, there is no single villain or architect responsible for these children's dental misery. Their parents are not the sole or even necessarily the major cause but, as this study suggests, children's distress results from the confluence of many factors in multiple contexts operating in concert in ways both obvious and subtle. While caregivers and families of young children may be an initial source of disease and of primary intervention for and prevention of oral disease in young children, parental ability to access professional dental services, including preventive services, for their children is highly dependent upon other contexts of care. It is imperative, then, that investigation of children's oral health status and attempts to reduce oral health disparities be investigated more broadly and interventions be undertaken in more than one context of care at a time.

## Conclusion

How exactly these identified contexts combine to create unmet treatment needs and prevent children from timely access to oral health care urgently warrants further theoretical as well as empirical investigation. Several conceptual models exist [30-32]. To date, however, little empirical research has examined the relationships among influences on children's oral health status identified in these conceptual models [30-32] or on the contexts in which such influences are manifest and interact. The ethnographic study presented here is just such an empirical examination.

Fisher-Owens and colleagues [31] present a useful conceptual model for moving research on children's oral health beyond primarily examining what is happening in the oral cavity or family to incorporating influences from other societal sectors as well. This conceptual model was developed from past research findings as well as insights and theories derived from the fields of population health and social epidemiology, fields that have recently developed multilevel, holistic approaches to analyze complex and interactive causes of children's health problems. Fisher-Owens' model identifies a variety of factors – genetic and biologic, social and physical environments, health behaviors, and dental and medical care – that influence oral health outcomes at the level of individual child, family, or community (including the state). Moreover, it recognizes that these influences change over time as a child matures and undergoes normal, expected life-course development and transitions. In this model, significant areas of influence affecting children's oral health status were identified, such as "Health Behaviors, Practices, and Coping Skills of Family", "Social Environment," "Social Capital," "Culture," "Community Oral Health Environment," "Physical Environment (including fluoridation)," "Use of Dental Care," "Dental Insurance status:" "Health Care System Characteristics" and "Dental Care System Characteristics" – influences that can easily be fit into the four intersecting contexts of care discussed in this paper. Another useful framework for understanding the findings presented in this article is Stokols' Socio-ecological model [32], although the dynamic interconnections between levels is not as well explicated as in the present report.

Overall, this paper demonstrates how diverse contexts and forms of dynamic influence interconnect to create and sustain the poor oral health status of young Latino children in impoverished rural communities. These influences comprise the thwarting of some efforts by parents to access quality care for their children, the constraining of some clinical options for dentists who provide services to indigent patients, and the unfortunate outcomes of policy and fiscal regulations that inadvertently encourage financial managers to distribute sparse resources in ways that are not as effective or equitable as they could be.

## Note

**1. **The terms 'Latino' and 'Hispanic' each carry particular demographic, historical and socio-political connotations [66]. A survey conducted by the Pew Hispanic Center/Henry J. Kaiser Family Foundation [67] found that in California in 2004 approximately half the population (51%) had no strong preference with respect to being called either 'Latino' or 'Hispanic.' We chose to use Latino, indicating that while the population in our study site originated mainly in Mexico, our sample also included people from several other Central American countries.

## Abbreviations Used

ECC – Early childhood caries; US – United States; WIC – federally-supported Women, Infant & Children nutrition services

## Competing interests

The author(s) declare that they have no competing interests.

## Authors' contributions

JCB developed the basic ideas for the main (NIDCR-funded) study, wrote the grant and received funding, and participated fully in its design, coordination and execution; hired and supervised all research staff and undertook all official management and administrative activities; helped develop the research protocols and codes; assisted with data analyses; wrote this manuscript. JCB also submitted and managed the UC Mexus-supported sub-study and reviewed its findings. SBH conceived the UC Mexus-supported sub-study and participated in its design, coordination and execution. She undertook the fieldwork and data collection for both the main (NIDCR) and the sub-study (UC Mexus-supported study), supervised field staff (for both the NIDCR and the UC Mexus studies); coded data and did analyses; wrote other manuscripts; reviewed and commented extensively on drafts of this manuscript. Both authors read, reviewed and approved the final manuscript.

## Pre-publication history

The pre-publication history for this paper can be accessed here:


